# Cultivable marine fungi from the Arctic Archipelago of Svalbard and their antibacterial activity

**DOI:** 10.1080/21501203.2019.1708492

**Published:** 2019-12-27

**Authors:** Ole Christian Hagestad, Jeanette H. Andersen, Bjørn Altermark, Espen Hansen, Teppo Rämä

**Affiliations:** a Marbio, The Norwegian College of Fishery Science, Department at Faculty of Biosciences, Fisheries and Economics, UiT The Arctic University of Norway, Tromsø, Norway; b The Norwegian Structural Biology Centre (NorStruct), Department of Chemistry, Faculty of Science and Technology, UiT the Arctic University of Norway, Tromsø, Norway

**Keywords:** Agar plug diffusion assay, Barents Sea, bioactivity, DNA barcoding, Lulworthiales, molecular phylogeny

## Abstract

During a research cruise in 2016, we isolated fungi from sediments, seawater, driftwood, fruiting bodies, and macroalgae using three different media to assess species richness and potential bioactivity of cultivable marine fungi in the High Arctic region. Ten stations from the Svalbard archipelago (73–80 °N, 18–31 °E) were investigated and 33 fungal isolates were obtained. These grouped into 22 operational taxonomic units (OTUs) using nuc rDNA internal transcribed spacer regions (ITS1-5.8S-ITS2 = ITS) with acut-off set at 98% similarity. The taxonomic analysis showed that 17 OTUs belonged to Ascomycota, one to Basidiomycota, two to Mucoromycota and two were fungal-like organisms. The nuc rDNA V1-V5 regions of 18S (18S) and D1-D3 regions of 28S (28S) were sequenced from representative isolates of each OTU for comparison to GenBank sequences. Isolates of Lulworthiales and Eurotiales were the most abundant, with seven isolates each. Among the 22 OTUs, nine represent potentially undescribed species based on low similarity to GenBank sequences and 10 isolates showed inhibitory activity against Gram-positive bacteria in an agar diffusion plug assay. These results show promise for the Arctic region as asource of novel marine fungi with the ability to produce bioactive secondary metabolites with antibacterial properties.

## Introduction

Terrestrial habitats have been extensively explored for fungi, resulting in discoveries of species able to produce antibiotic, anticancer, antifungal, and immunomodulating compounds, among others (Bills and Gloer 2016). However, marine fungi have, to a large degree been neglected by bioprospectors and remain a potential source of novel compounds (Imhoff 2016). This is reflected by the large number of undescribed species that frequently appear in environmental samples and the chemical diversity that is still being uncovered (Richards et al. 2011, 2015; Jeffries Thomas et al. 2016; Ji and Wang 2016; Rämä et al. 2016; Hassett et al. 2017; Reich and Labes 2017). Studies on bioactive compounds from marine fungi have mainly focused on fungi from tropic and temperate regions. These fungi are most frequently isolated from specific hosts, such as mangrove trees, sponges, algae, and corals (Debbab et al. 2010; Jones and Pang 2012; Thatoi et al. 2013; Yarden 2014; Bajpai 2016; Pang et al. 2016; Sridhar 2017). However, the Arctic remains underexplored compared to other regions with a relatively low number of studies on marine fungi (Shearer et al. 2007; Imhoff 2016; Tisthammer et al. 2016; Rämä et al. 2017; Hassett et al. 2019).

The low number of studies on Arctic marine fungi might be explained by varying sea ice conditions and remoteness of the Arctic making it difficult and expensive to access study locations. A couple of studies have explored the fungal diversity in the polar regions using both cultivation and metagenomics approaches. Metabarcoding studies show a large marine fungal diversity, reporting hundreds to thousands of OTUs (Zhang et al. 2015; Comeau et al. 2016; Rämä et al. 2016; Tisthammer et al. 2016). Of these, 10–30% remain unidentified to order level within the Fungi kingdom, indicating a high-level richness of putatively undescribed species in the Arctic. Comeau et al. (2016) were also able to show that the frequency of certain fungal sequences increased from temperate to Arctic waters, indicating that some species or strains are specifically adapted to the Arctic environment.

Even though metabarcoding is an efficient tool for studying taxonomic diversity, it reveals nothing about the ability of a fungi to produce secondary metabolites. A review of studies on marine fungi in the Arctic found that only 13 studies investigated cultivable marine fungi from the Arctic, none of these investigated potential secondary metabolites from the fungi (Rämä et al. 2017). A review of bioactive fungal isolates from polar regions found that most studies were from soil samples, freshwater ponds and lakes, or exclusively Antarctic marine sources; no papers were found reporting bioactivity from any Arctic marine fungal sources (Lo Giudice and Fani 2016). We have found three papers reporting metabolites from Arctic marine fungi, all of them isolated from marine sediments. *Trichoderma* sp. strain MF106 from the Greenland Sea showed activity towards *Staphylococcus epidermidis* (Wu et al. 2014). Metabolites isolated from *Tolypocladium* sp. and *Mortierella* sp. from Frobisher Bay, Nunavut, Canada did not exhibit any significant antibacterial or cytotoxic activity (Grunwald et al. 2016, 2017). These are the only papers reporting of bioactivity from Arctic marine fungal sources. With results from only three isolates published in scientific articles, there is a severe lack of knowledge about bioactivity of Arctic marine fungi.

The aim of this study was to isolate and identify fungi from different substrates in the Arctic. In addition, the potential of the isolates to produce secondary metabolites was assessed by examining their antibacterial activity against five common human pathogens in an agar plug diffusion assay.

## Materials and methods

### Sampling and isolation of marine fungi

Samples were collected during a bioprospecting cruise with R/V Helmer Hansen around Svalbard in September 2016 (see Supplementary figure and Table 1 for exact locations and metadata). In addition to fruiting bodies, four different microhabitats were sampled: marine macroalgae, driftwood, sediments, and seawater. Marine macroalgae were collected from intertidal sites and were handpicked into sterile bags. Algal samples were surface sterilised by cutting the material into pieces of approximately 1 × 1 cm and dipping them into 70% ethanol for 30 seconds before rinsing them in autoclaved MilliQ water. Two to three pieces were then plated onto one 0.4% malt extract agar prepared with artificial seawater (ASMEA), corn meal agar in artificial seawater (ASCMA) and 1% dried and homogenised *Ascophyllum nodosum* in artificial seawater (1.0ASAsco) plates containing 30 µg/mL streptomycin and 30 µg/mL tetracycline. Driftwood samples, obtained from the intertidal zone or bottom trawl, were prepared following the procedure from Rämä et al. (2014) by removing the surface of the wood with a sterile knife and then cutting out 0.5 × 1 cm pieces. Two to three pieces were plated on each type of plate. Fruiting bodies (sporocarps) from driftwood were moistened with seawater and let dry until spores were discharged and then transferred to agar plates. Sediment samples were collected using a Van Veen grab. The surface of the material in the grab was removed using a sterile knife and sediment was scooped using a sterilised knife into a Petri dish that was closed immediately afterwards. An inoculation loop was used to streak out the sediment onto the agar plates. *In-situ* culturing plates were made by pouring inoculated agar into a mould and covering it with barrier membranes which only allows small molecules to pass through. These plates were inoculated in a seawater pool with constant seawater flow on the deck of the boat during the cruise. The cell suspension for the *in-situ* agar inoculum was sampled in a sterile Duran flask on a cruise to approximately the same area (76°15′27.5″N, 29°51′35.0″E and 5 m depth) in May 2016, was stored at +2°C and used undiluted in September. This method is inspired by the technique described by Nichols et al. (2010) and Berdy et al. (2017).

Once fungal mycelium was visible and had grown out from the substrate it was transferred to a new agar plate until an axenic culture was obtained. The subculture-media did not contain antibiotics. All incubations were done at 4–10.

### Sanger sequencing and OTU clustering

A small piece of agar containing mycelium was transferred to an Eppendorf tube and 100 µl of autoclaved MilliQ water was added, followed by vigorous vortexing. For the PCR reaction, 1 µl of this material was used as a template. DreamTaq Green PCR Master Mix (2X) was used together with the different primer sets (Supplementary table 2). The barcode region sequenced was the nuc internal transcribed spacer rDNA (ITS1-5.8S-ITS2 = ITS) using primers ITS5/ITS4 (White et al. 1990), with a backup set ITS3(White et al. 1990)/LR0Ri (Inverted LR0R sequence) for ITS2.

Successful PCRs were purified using either QIAquick PCR purification kit, ExoSAP-IT or A’SAP PCR clean-up treatment according to the manufacturer’s manual. The purified PCR products were then prepared for two directional Sanger sequencing reaction using BigDye3.1 and a PCR program of 30 cycles at 95 °C for 30 seconds, 47 °C for 10 seconds and 72 °C for 1 minute. The sequencing was performed by the sequencing platform at the University Hospital of North Norway utilising Applied Biosystems 3130xl Genetic Analyser (Life Technologies/Applied Biosystems). The returned chromatograms were imported into Geneious v10.2.3 (https://www.geneious.com/), trimmed to 0.05 error probability, primer regions removed, assembled into consensus sequences and proofread according to guidelines proposed by Nilsson et al. (2012).

ITS sequences were trimmed to the same start and end before they were aligned using MAFFT plugin v7.388 in Geneious v10.2.3 using E-INS-I algorithm with scoring matrix PAM200 (Katoh et al. 2002; Katoh and Standley 2013). The aligned sequences were then used to cluster sequences into OTUs using MOTHUR v.1.35.1 at 98% ITS similarity cut-off (Schloss et al. 2009). The full length sequences of each cluster were queried against Rfam for intron detection (Bateman et al. 2017; Kalvari et al. 2018). A list of the isolates and OTU division can be found in Supplementary table 3.

Representative sequences for each OTU were sequenced for two additional barcode regions. The nuc rDNA covering V1-V5 regions of 18S (18S) using primers NS1/NS4 (White et al. 1990), and nuc rDNA covering D1-D3 regions of 28S (28S) using primers LR0R/LR5 (Vilgalys and Hester 1990; Rehner and Samuels 1994).

The PCR reaction parameters for ITS and 28S were an initial denaturation at 95 °C for 5 minutes, followed by 35 cycles of denaturation at 95 °C for 30 seconds, annealing at 47 °C for 30 seconds, elongation at 72 °C for 1 minute. A final elongation step of 72 °C for 10 minutes was carried out followed by a hold at 4 °C indefinitely. 18S amplification only differed in annealing temperature of 42 °C. Success of the amplification was checked by gel electrophoresis of the samples on 1% Tris-Borate-EDTA agar gel infused with 0.1‰ GelRed. A list of accession numbers for sequences generated in this study can be found in Supplementary table 4.

### OTU identification and phylogenetic analyses

Representative sequences of each OTU were submitted to GenBank for a BLAST search with an ENTREZ query string limiting hits to fungal and fungal-like origin and excluding uncultured, metagenomic, and environmental samples to limit the numbers of hits to sequences of known origin of relative high quality (Souvorov et al. 2006; Raja et al. 2017). Sequences for each locus were downloaded from GenBank and aligned. If available, type strains with sequences from all marker regions (18S, ITS and 28S) were preferably downloaded. Strains were only included if two of the three loci were available in GenBank. A list of reference sequence accession numbers is listed in Supplementary table 5.

Alignment of the sequences were done in Geneious 10.2.3, using MAFFT v.7.388 for each separate barcode region before they were concatenated to a single sequence. The sequences were aligned using different algorithms for different markers, E-INS-I for ITS and 28S, and G-INS-I for 18S. E-INS-I considers multiple conserved domains interspaced with long gaps or regions harder to align. G-INS-I considers a global homology between the sequences. The scoring matrices used were PAM200 for ITS, and PAM100 for 18S and 28S to account for difference in sequence similarity.

The multiloci dataset of 3148 nt was partitioned using PartitionFinder v2.1.1 (Lanfear et al. 2017) and jModelTest v2.1.10 (Posada 2008) was used to find the best substitution model and priors selected by the corrected Akaike information criterion (AICc) and Bayesian information criterion (BIC). The three suggested partitions were 18S and 5.8S, ITS1 and ITS2, and 28S. The suggested model GTR+I + G for 18S and 28S, and SYM+I + G for ITS was changed to GTR+G to avoid simultaneous optimisation of both gamma and the proportion of invariant sites as these parameters correlate strongly (Jia et al. 2014). Instead, six gamma categories was used to accommodate for slowly evolving sites. A prior mean branch length, $\bar{brl}$ was first estimated using maximum likelihood. The prior mean was used to set the parameters for the exponential prior exp (λ) where $\lambda =\frac{1}{\bar{brl}}$.

The resulting alignments, partitions and models were used for Maximum likelihood and Bayesian tree construction using RAxML v8.2.11 in Geneious (Stamatakis 2014) and MrBayes v.3.2.6 (MPI parallel version with Beagle) (Ronquist et al. 2004, 2012). RAxML was run with the following settings: Substitution model GTR gamma, rapid bootstrapping and search for best scoring ML tree with 2000 bootstrap replicates.

The nexus file with alignments and MrBayes command block can be found in the supplementary data. Tracer v1.7.1 was used to assess the convergence of the runs (Rambaut et al. 2018). The optimisation of proposal mechanisms was done according to Ronquist et al. (2009). The analysis was run until the standard deviation of the split frequency stabilised at a value or dropped below 0.05. The command blocks used and resulting trees have been deposited to Mendeley Data and are available at http://dx.doi.org/10.17632/52dkhy5xsb.1 (Hagestad et al. 2019).

### Agar plug diffusion assay

For the diffusion assay, 5 or 6 agar plugs (diameter 8 mm) from different samples with actively growing mycelium were placed evenly spaced in an empty petri dish. Mueller-Hinton (MH) or brain heart infusion (BHI)-agar was infused with overnight culture (100 µL in 100 mL) of *Staphylococcus aureus* (ATCC 25923), *Streptococcus agalactiae* (ATCC 12386), *Enterococcus faecalis* (ATCC 29212), *Pseudomonas aeruginosa* (ATCC 27853) or *Escherichia coli* (ATCC 25922), the latter two using BHI-agar. The inoculated agar was poured on the petri dishes until the agar reached the height of the plugs. The plates were then incubated for approximately 24 hours at 37 °C and the diameter of inhibition zones was measured.

## Results

### Richness and phylogenetic diversity of isolated fungi

**Figure 1.**
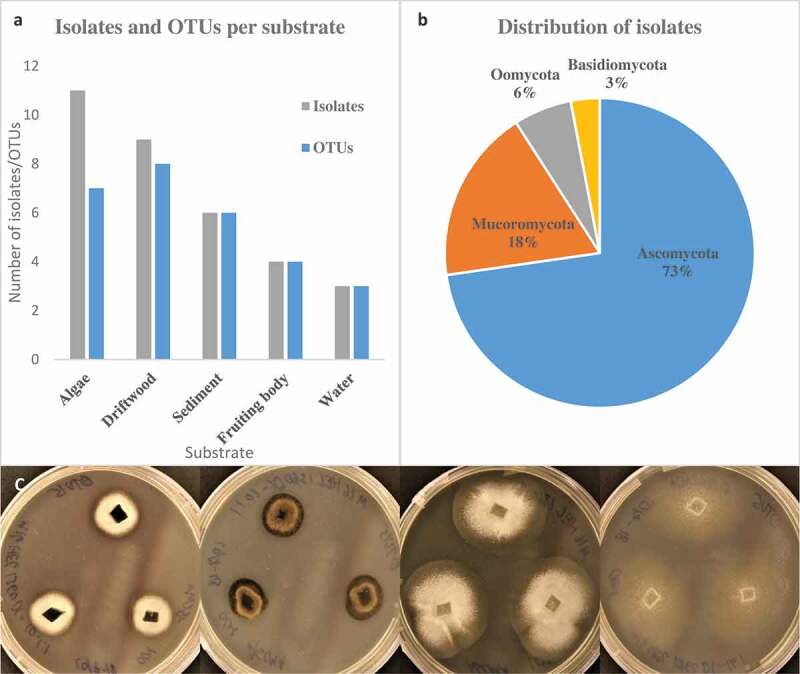
(a) Overview over substrate source and number of isolates and OTUs. (b) Phylum level distribution of isolates. (c) Illustration of two different isolates on two media. From right: *Mytilinidion* sp. OTU15 on ASCMA and ASMEA, *Pseudeurotium* sp. OTU5 on ASCMA and ASMEA.

A total of 197 isolates were analysed from algae, sediment, driftwood, fruiting bodies, and seawater. Sequences that failed to assemble or were truncated were excluded from further analysis. Most of the sequences had >80% high quality base calls. In cases where samples had identical sequences and came from the same isolation plate, only one was selected as an isolate to prevent isolation of the same fungus several times. This finally resulted in 33 different isolates. Eleven isolates came from littoral macroalgae, followed by benthic driftwood (9), benthic sediments (6), fungal fruiting bodies from benthic driftwood (4), and *in-situ* cultures from seawater (3) (Figure 1(a)).

**Figure 2.**
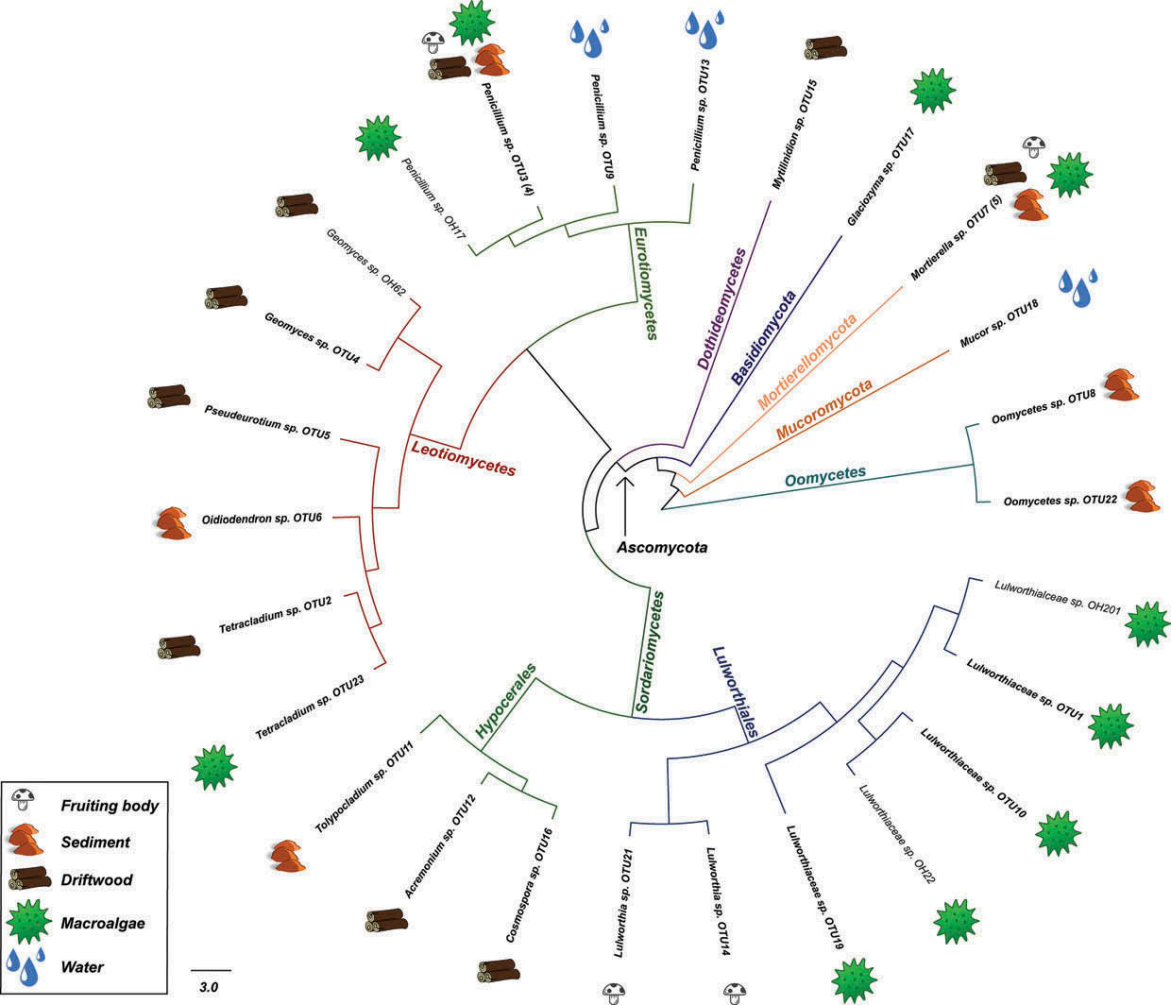
Graphical representation of the 33 isolates in this study based on a Maximum likelihood tree constructed from the ITS sequences. Taxonomic ranks are colour coded in branches. Substrates are marked with icons along the edge after isolate name. Representative isolates names are in bold and numbers of identical sequences represented by the entry in brackets. Scale bar show nucleotide substitutions per site.

Clustering of similar ITS sequences grouped the isolates into 22 unique OTUs (Figure 1(a)). Seven OTUs were found in algae, eight in driftwood, six in sediments, four in fruiting bodies, and three in water. The OTUs were generally found in only one sample and only one substrate with a few exceptions. *Penicillium* sp. OTU3 and *Mortierella* sp. OTU7 were found in algae, driftwood, fruiting bodies, and sediments, indicating a wide substrate preference. The distribution of the specific OTUs and isolates among the substrates can be seen in Figure 2. The most speciose substrate was driftwood, which yielded 8 OTUs. Some OTUs were found in multiple locations, e.g. Lulworthiaceae sp. OTU1 and *Geomyces* sp. OTU4. A complete list of representative isolates is shown in Supplementary table 4.

The isolates had some identifiable morphological traits. All but one OTU were filamentous. One basidiomycete, *Glaciozyma* sp. OTU17, was able to change between a filamentous and a unicellular yeast form. *Mucor, Penicillium* spp., *Oidiodendron*, and *Tolypocladium* produced spores, presumably conidiospores. *Lulworthia* sp. OTU14 and OTU21 were confirmed to belong to *Lulworthia* based on isolated ascospores from fruiting bodies on driftwood that germinated and were used to establish mycelial cultures. Isolates were observed to have different morphological characteristics (colour, growth pattern) on different media during cultivation (Figure 1(c)).

The isolates spanned four divisions (three fungal), seven classes, 10 orders, 14 families, and 15 genera. The pairwise identity with reference sequences from GenBank ranged from 78.9–100%, with identity being highest in species also known from terrestrial sources (*Penicillium, Mucor, Geomyces* and *Mortierella*) and lowest in marine species and poorly studied genera (Lulworthiales and Oomycota). Based on identity in GenBank, nine isolates seemed to represent undescribed species with ITS similarity of less than 96% and LSU similarity ranging from 89–99% (Supplementary Table 4). Most of the isolates belonged to Ascomycota (73%), followed by Mucoromycota (18%), fungal-like organisms (Oomycota) (6%), and finally Basidiomycota (3%) (Figure 1(b)).

**Figure 3.**
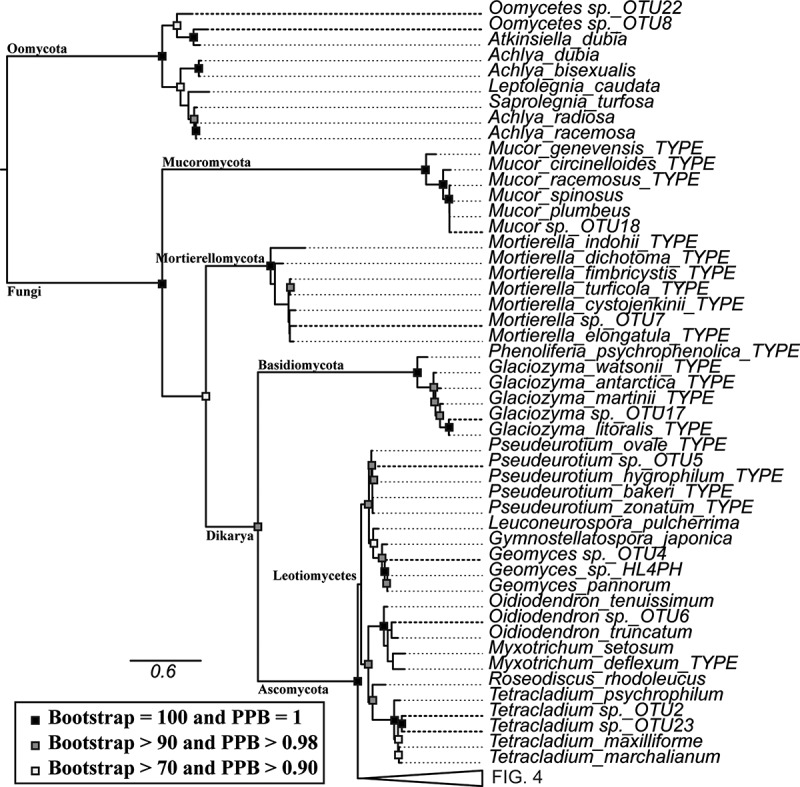
Taxonomic placement of representative isolates used in the study (bold), showing portion close to the base of the tree. RAxML tree with bootstrap and posterior probability support values where nodes are identical with Bayesian analysis marked with filled boxes. Phyla, order, class and family is noted on respective branches. Accession numbers of reference sequences can be found in Supplemental Table 5.

**Figure 4.**
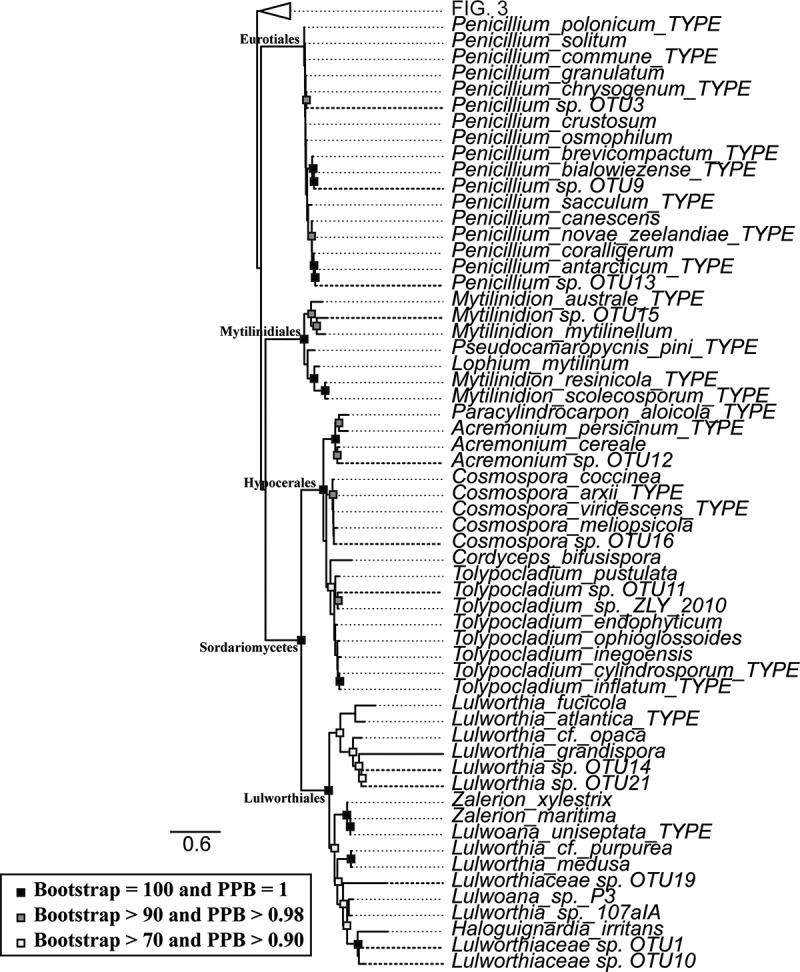
Taxonomic placement of representative isolates used in the study (bold), with base of the tree collapsed. RAxML tree with bootstrap and posterior probability support values where nodes are identical with Bayesian analysis marked with filled boxes. Phyla, order, class and family is noted on respective branches. Accession numbers of reference sequences can be found in Supplemental Table 5.

Both maximum likelihood and Bayesian inference arrived at similar topologies and relative branch lengths using all three sequenced loci, contrasting in nodes at the class level and placement of terminal nodes in *Oidiodendron, Penicillium, Cosmospora*, and *Tolypocladium* (Figures 3 and 4). The deep nodes were generally well supported as well as nodes closer to the terminal branches.

In most cases the OTUs were placed within monophyletic clades, but there were a few exceptions. The fungal-like organisms in this study were placed outside the referenced genera within Saprolegniales. Lulworthiaceae sp. OTU19, Lulworthiaceae sp. OTU10, and Lulworthiaceae sp. OTU1 were placed outside of the genus *Lulworthia*, together with sequences of *Lulworthia, Zalerion, Lulwoana*, and *Haloguignardia* indicating that more thorough work on phylogeny of marine fungi within Lulworthiales is needed for proper placement.

### Antibacterial activity

Ten of the 20 tested isolates showed activities against at least one human pathogenic bacterium (Table 1). Activities were observed against the Gram-positive bacteria, *S. aureus, E. faecalis*, and *S. agalactiae*. Eurotiomycetes and Leotiomycetes gave the strongest antibacterial activity. *Acremonium* sp. OTU5 and *Mortierella* sp. OTU7 of Sordariomycetes and Mortierellomycetes, did also give strong antibacterial activity. Most of the growth inhibition was observed against *S. aureus* and *S. agalactiae*. However, *Pseudeurotium* sp. OTU5 and *Geomyces* sp. OTU4 were active against *E. faecalis. Penicillium* sp. OTU13 was noted for having high and specific activity against *S. agalactiae*, many times higher than any other isolate. *Mortierella* sp. OTU7 was the only isolate in this study outside of Ascomycota with activity towards the pathogenic bacteria. The activity against *S. agalactiae* and *E. faecalis* were only present when cultured on ASCMA, with the exception of *Penicillium* sp. OTU13 which had activity towards *S. agalactiae* when cultured on ASMEA.

**Table 1.** Results from agar plug diffusion assay given as average inhibition zone diameter in mm. Average is based only on active plugs. Plugs used were 8 mm in diameter. Numbers in superscript are numbers of active plugs out of total plugs tested for a specific isolate on a specific media. There was no activity against gram negative bacteria (not shown).

	ASCMA^a^			ASMEA^b^		
Name	*S. aureus*	*E. faecalis*	*S. agalactiae*	*S. aureus*	*E. faecalis*	*S. agalactiae*
Lulworthiaceae sp. OTU1	−	−	−	−	−	−
Lulworthiaceae sp. OTU10	−	−	−	−	−	−
*Lulworthia* sp. OTU14	−	−	−	−	−	−
*Lulworthia* sp. OTU21	−	−	−	−	−	−
Lulworthiaceae sp. OTU19	−	−	−	n.d.	n.d.	n.d.
*Acremonium* sp. OTU12	−	−	9^1/3^	10.5^2/4^	−	−
*Cosmospora* sp. OTU16	−	−	−	9^1/4^	−	−
*Tolypocladium* sp. OTU11	−	−	−	−	−	−
*Oidiodendron* sp. OTU6	−	−	−	−	−	−
*Pseudeurotium* sp. OTU5	12^1/3^	10^1/3^	−	−	−	−
*Tetracladium* sp. OTU23	−	−	−	−	−	−
*Tetracladium* sp. OTU2	−	−	−	9^2/5^	−	−
*Geomyces* sp. OTU4	14^3/4^	12^1/4^	10^1/4^	12.3^3/3^	−	−
*Penicillium* sp. OTU3	17^1/4^	−	13^2/4^	9^3/3^	−	−
*Penicillium* sp. OTU9	11^1/3^	−	−	11.7^3/3^	−	−
*Penicillium* sp. OTU13	12^1/3^	−	12.5^2/3^	9^3/3^	−	28^3/3^
*Mytilinidion* sp. OTU15	−	−	−	11.3^3/3^	−	−
*Glaciozyma* sp. OTU17	−	−	−	−	−	−
*Mortierella* sp. OTU7	12.5^2/3^	−	−	−	−	−
*Mucor* sp. OTU18	−	−	−	−	−	−

**^a^**Corn meal agar in artificial seawater

**^b^**0.4% Malt extract agar in artificial seawater

n.d. = no data | − = no activity

## Discussion

Fungi remain one of the most understudied group of microbial organisms in the ocean and they represent a good source for novel biochemistry (Liberra and Lindequist 1995; Richards et al. 2011; Berlinck 2013; Wang et al. 2015). The relatively few studies on cultivable fungi undertaken in the Arctic underlines the importance of mapping and exploring the species richness and capabilities of marine fungi in this region (Rämä et al. 2017; Luo et al. 2019). The present study provides the first insight into the bioactivity of cultivable marine fungi isolated from diverse substrates from cold waters of the Arctic region.

We found 22 unique OTUs (98% ITS similarity) from the 33 isolates originating from substrates collected at 10 stations. Ascomycota was the most abundant division, accounting for 73% of the isolates. The relative abundance of Ascomycota compared to other divisions is similar to what has been described in other culture-dependent studies on marine fungi from for example driftwood and sediments (Bubnova 2010; Pang et al. 2011; Nagano and Nagahama 2012; Rämä et al. 2014; Blanchette et al. 2016; Bubnova and Nikitin 2017). The number of isolates obtained in culture dependent studies varies considerably, from as low as eight to as high as 577 (Singh et al. 2012; Rämä et al. 2014; Blanchette et al. 2016). However, direct comparison of the different articles is difficult due to differences in study focus, methods, isolation substrate, media, and geographical area.

It is interesting to note that although fruiting bodies collected from driftwood belonged to the Lulworthiales, none of the driftwood samples themselves yielded Lulworthiales isolates. This could be due to low number of driftwood samples, or that other species outgrew Lulworthiales on the isolation plate. Generally, isolates of Lulworthiales grew slowly compared to other isolates.

Despite the modest number of isolates described in this study, the species richness is high, spanning four different phyla and 10 different orders, providing isolates with both high and low similarities to known sequences in GenBank. Based on sequence similarity compared to GenBank sequences of the three loci, nine of the isolates seem to be novel species. Whether the novel sequences represent novel species remains unclear, as many species and old type specimens are not properly sequenced for the different rDNA loci used in this paper. Most of the reference sequences in GenBank have been deposited after 2007 and most species have been added after 2014. Curated fungal databases have few sequences of marine origin, and only 12% of all marine fungal genera are represented in RefSeq (Hassett et al. 2019). Other databases have similar deficiencies of marine representation and provides an equally poor source of identification and comparison of the isolates. When isolates have a poor hit score, identification becomes much more difficult as it is hard to determine the proper taxa. However, with multiple loci it is possible to ascertain a proper taxonomic placement, at least to family or genus level by comparing the similarity of the loci to reference sequences.

During manual OTU control after running MOTHUR it was seen that *Tetracladium* OTU2 and OTU23 were included in the same OTU, despite a 285 bp insert at the end of 18S rDNA in OTU23. This insert differentiated OTU23 markedly from OTU2, although the ITS sequences themselves had only five parsimony-informative sites over 556 bp, making them more than 99.1% similar. A similar insertion of approximately 335 bp was found in *Mytilinidion* sp. OTU15 in the same region. These 18S rDNA inserts of about 300 bp were determined to be group 1 introns by Rfam and are known to occur at the end of 18S rDNA (Rogers et al. 1993; Gargas et al. 1995; Hibbett 1996; Taylor et al. 2016). The ITS sequences of *Lulworthia* sp. OTU14 and OTU21 were only 96.3% similar, but they had the same sequence gaps, and differed only in some G-A and C-T conversions that are common polymerase errors and could represent the same OTU when 18S and 28S rDNA sequences are considered (Potapov and Ong 2017). In addition to this, the ribosomal DNA exists in multiple copies with inherent sequence variations and can also lead to ITS sequence variation within a single specimen (Simon and Weiß 2008). Our observations support the conclusion that ITS is not a perfect barcoding region alone and highlights that ITS similarity and dissimilarity can be misleading and that manual control is important where applicable (Schoch et al. 2012).

Both maximum likelihood and Bayesian phylogenetic trees provided a consensus for the classification of the sequences to the determined level of taxonomy. The long terminal branches within Lulworthiales, Mytilinidiales and fungal-like organism clades indicate novel species of poorly studied families or poor availability of reference sequences.

There were some higher rank taxa that were not recovered in this culture-dependent study, such as the phylum Chytridiomycota and yeast-like fungi in Ascomycota (Saccharomycetales), although these are known to frequently occur in the Arctic marine environment (Zhang et al. 2015; Hassett et al. 2017). The reason why these groups remained unrecovered is likely methodological, as different kinds of isolation and culturing techniques or media are needed to culture these (Sparrow 1960; Kutty and Philip 2008). We used methods that are known to capture a broad diversity of filamentous fungi in Dikarya and other taxa such as some fungal-like Oomycota. Testing the recovered diversity in antibacterial bioassays allowed us to give a preliminary account of the antibacterial activity of Arctic marine fungi.

### Antibacterial activity

The agar plug diffusion assay was chosen for bioactivity screening as it is a relatively simple method to perform and can give an indication of isolates producing antibacterial compounds. In addition, fungi growing on solid media has been shown to produce higher amounts and more diverse bioactive molecules compared to liquid cultures (VanderMolen et al. 2013). The incubation period for the agar plug diffusion assay was relatively short, only 24 hours, possibly not allowing all fungi to properly respond to the presence of the bacteria. The incubation temperature (37 °C) was also high compared to the temperatures these fungi exist at in nature (less than 10 °C). At lower temperatures, such as room temperature, fast growing fungi quickly overgrew the plate before the pathogenic bacteria could grow. However, the agar plug diffusion assay provides a way to detect constitutively expressed molecules or molecules that are expressed during stress.

The only isolate with activity against all Gram-positive bacteria tested was *Geomyces* sp. OTU4 when cultured on ASCMA. The *Penicillium* OTUs on the other hand, had the overall strongest antimicrobial activity, with activity towards *S. aureus* and *S. agalactiae*, supporting the evidence of some genera in Eurotiales as potent producers of antimicrobial compounds (Richards 2011). The different *Penicillium* OTUs also had different antimicrobial activities. Species from the same genus within *Penicillium, Aspergillus*, and *Fusarium* are known to have widely different metabolic profiles (Bladt et al. 2013; Frisvad 2014; Nesic et al. 2014; Hasan et al. 2015; Wang et al. 2015). Ten different *Penicillium* species have previously been challenged with different growth conditions in order to examine shifts in their metabolic profiles. The study showed that *Penicillium* species have highly variable secondary metabolite production, ranging from producing 4 to 34 different identified secondary metabolites. These metabolites were often species specific, among the identified metabolites only six were shared between some of the species, none were shared between all (Grijseels et al. 2017).

Another observation we made was that bioactivity changed over time. Even though a single isolate tested several times the same day provided the same inhibition zone against the same bacteria, the activity varied if tested a week, a month or several months later indicating that the expression changed over time. This difference in activity could also be due to difference in fungal biomass on the plugs as they had different times to grow between the samplings. This time-dependent difference in metabolism has been shown for different *Penicillium* species before (Khalil et al. 2014; Roullier et al. 2016).

Several of the tested OTUs showed altered antibacterial activity on the two different media. This was especially apparent in *Penicillium* sp. OTU13 that had an inhibition zone against *S. agalactiae* of 12.5 mm when cultured on ASCMA and 28 mm on ASMEA. On the other hand, the inhibition zone towards *S. aureus* decreased from 12 mm on ASCMA to 9 mm on ASMEA indicating that the activity towards *S. agalactiae* was specific. For some fungi, the alteration of growth media produced different selectivities in antibacterial activity, as seen with *Acremonium* sp. OTU12, with activity against *S. aureus* on ASCMA and on *S. agalactiae* on ASMEA. Grijseels et al. (2017) also reported that the species altered expression patterns when cultured in different culture media.

Bioactivity can be dependent on many factors such as culture medium, temperature, water activity, light, pH, salt content, culture time, and presence of small molecules (Sepcic et al. 2011; Hewage et al. 2014; Gubiani et al. 2016). Our results illustrate medium-dependent changes of bioactivity. This has also previously been shown by Overy et al. (2017) in a comprehensive study where the same ex-type strain of *Aspergillus aculeatus*, a halotolerant terrestrial fungus, produced distinct culture media-specific metabolites when grown with different osmotic pressures as well as laboratory (site) specific metabolites.

Some of the species isolated in this study have likely been isolated before in other studies if sequence similarity is considered. However, this does not mean that new discoveries from these isolates cannot be made. A different species, *Aspergillus flavus*, is a cosmopolitan species present all over the globe, and a thorough examination by Ramírez-Camejo et al. (2012) showed that the terrestrial and marine isolates of this species composed a single population. This, together with the results of Overy et al. (2017) shows that isolation of completely novel species is not necessary to uncover unique secondary metabolites, as these may be expressed just by culturing in a different laboratory environment. This highlights the importance of using the one strain, many compounds (OSMAC) approach to express secondary metabolites from cultured strains, and to use parameters from different habitats as inspiration for culture conditions.

This study shows that there is potential to make discoveries in the Arctic and that the research community should put more effort into identifying and characterising marine fungi and fungal metabolites from the Arctic to reduce the current knowledge gap.

## Supplementary Material

Supplemental Material

Supplemental Material

Supplemental Material

## Geolocation information

The approximate midpoint of the stations in this study is 78° 13′ 57.6″N, 27° 46′ 11.3″Ø. The individual stations can be seen in Supplementary Figure 1 and location data can be found in Supplementary Table 1.
