# Supplementary figures and images for: Cultivable marine fungi from the Arctic Archipelago of Svalbard and their antibacterial activity
Source: Mycology. 2019 Dec 27;11(3):230–42. doi: 10.1080/21501203.2019.1708492 (PMC7534220; doi:10.1080/21501203.2019.1708492)

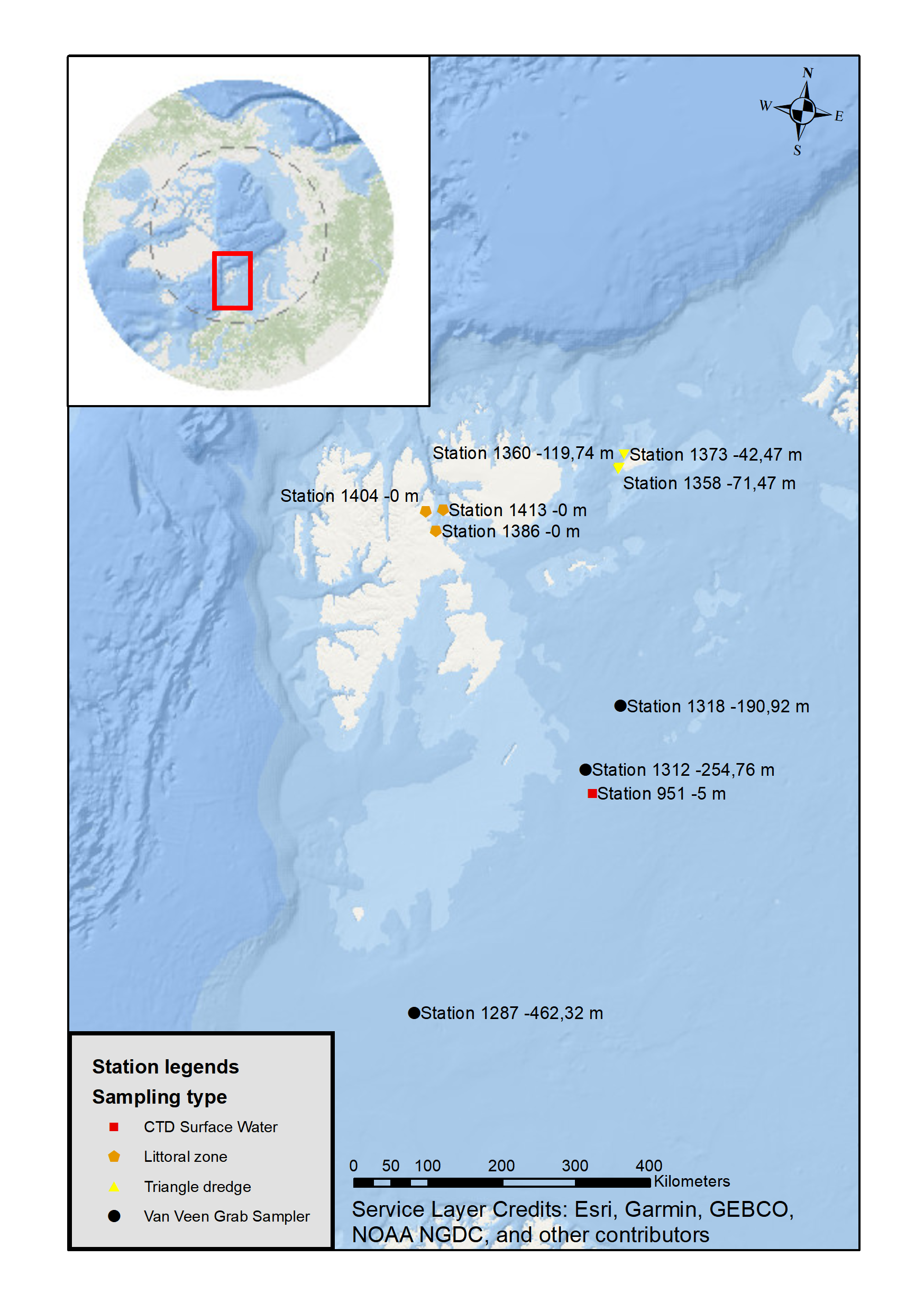

Supplement: Supplemental Material [file TMYC_A_1708492_SM2308.tif]
