# Supplementary material for: Cultivable marine fungi from the Arctic Archipelago of Svalbard and their antibacterial activity
Source: Mycology. 2019 Dec 27;11(3):230–42. doi: 10.1080/21501203.2019.1708492 (PMC7534220; doi:10.1080/21501203.2019.1708492)
Supplement: Supplemental Material [file TMYC_A_1708492_SM2307.docx]

# Supplementary Tables

**Supplementary Table 1.** Location and metadata of stations sampled in this study.

**Supplementary Table 2.** Primer sequences and references used in this study

**Supplementary Table 3.** Isolates obtained in this study with clustering to OTUs

**Supplementary Table 4.** Representative isolates with accession numbers, isolate ID, Rfam accession and pairwise similarity to GenBank sequences.

**Supplementary Table 5.** Reference sequences with accession used in alignment.
